# Involvement of *CitCHX* and *CitDIC* in Developmental-Related and Postharvest-Hot-Air Driven Citrate Degradation in Citrus Fruits

**DOI:** 10.1371/journal.pone.0119410

**Published:** 2015-03-04

**Authors:** Qiong Lin, Shaojia Li, Wencheng Dong, Chao Feng, Xueren Yin, Changjie Xu, Chongde Sun, Kunsong Chen

**Affiliations:** Laboratory of Fruit Quality Biology, The State Agriculture Ministry Laboratory of Horticultural Plant Growth, Development and Quality Improvement, Zhejiang University, Hangzhou, 310058, P. R. China; Washington State University, UNITED STATES

## Abstract

Citrate is the predominant organic acid associated with taste in citrus fruit. Although citrate metabolism has been widely studied in recent years, the potential contributions of transport proteins to citrate content remain unclear. In the present study, high-acid citrus fruit Gaocheng (‘GC’, *Citrus sp*.) and low-acid citrus fruit Satsuma mandarin (‘SM’, *Citrus unshiu* Marc.) were selected for study, and the degradation of citrate was deduced to be the main cause of the difference in acidity in fully mature fruits. RNA-seq analysis was carried out on ‘GC’ and ‘SM’ fruit samples over the same time course, and the results indicated that citrate degradation occurred mainly through the glutamine pathway, catalyzed by *CitAco3-CitGS2-CitGDU1*, and also two transport-related genes, *CitCHX* and *CitDIC*, were shown to be associated with citrate degradation. These results were confirmed by real-time PCR. In postharvest ‘GC’ fruit, the expressions of these two transport-related genes were induced by 2-fold under hot air treatment, accompanied by a reduction of 7%-9% in total acid degradation. Transient expression of *CitCHX* and *CitDIC* in tobacco leaves was performed, and the citrate content was reduced by 62%, 75% and 78% following *CitCHX*, *CitDIC* and *CitCHX* plus *CitDIC* treatments, respectively, as compared with expression of an empty vector. Overall, these data indicated that two transport proteins, *CitCHX* and *CitDIC*, are not only involved in citrate degradation during fruit development, but also involved in postharvest hot air triggered citrate reduction.

## Introduction

Organic acids are involved in central metabolisms and stress tolerance in plants. The accumulation of organic acids in plant cells is the result of several interlinked processes that take place in different compartments of the cell and appear to be under the control of many factors. The enzymes evolved in each step of organic acid metabolism pathways have been characterized by decades of research with mutants and model plants. Citrate is synthesized by the condensation of oxaloacetic acid (OAA) and acetyl-CoA, catalyzed by citrate synthase (CS) [1]. The synthesis of OAA requires fixation of CO_2_, which is achieved through the carboxylation of phosphoenolpyruvic acid (PEP), catalyzed by phosphoenolpyruvate carboxylase (PEPC) [2]. Once citrate has been produced by the tricarboxylic acid (TCA) cycle, it can be degraded through one of three metabolic pathways, including the gamma-aminobutyric acid (GABA), glutamine and acetyl-CoA pathways. The most studied route is the GABA pathway, which leads to succinate synthesis [3]. The second is the acetyl-CoA pathway, which may occur during the fruit ripening [4], and leads to cleavage of citrate into OAA and acetyl-CoA catalyzed by ATP-citrate lyase (ACL) [5]. It has been reported that 2-oxoglutarate can be transformed into glutamate and then converted into glutamine and possibly utilized for thiamine biosynthesis [6]. According to previous research, the difference in acidity of various citrus fruit is mainly due to the degradation of citrate [7, 8], however, the critical steps controlling transportation of citrate remain unknown.

Most research related to organic acid transport proteins has been conducted on root cells, such as *ZmALMT2*, *AtMATE*, *TaMATE1B* and *FRD3*, have been characterized [9–12]. However, the transport proteins identified in these studies may not only function specifically in root cell, but also participate in organic acid distribution and pH regulation in other organs and tissues. For instance, *OsFRDL1* is a citrate transporter localized in the pericycle cells, which is necessary for efficient translocation of iron (Fe) to the shoot as a Fe-citrate complex [13]; *HvALMT1* functions as a malate channel to facilitate malate transport in stomatal function and expanding cells [14, 15]; tonoplast-localized proteins *NHX1* and *NHX2* are involved in endosomal pH regulation and salt tolerance in *Arabidopsis* [16]. Thus, the content of organic acids in plants is not only controlled by biosynthesis and degradation, but is also affected by numerous transport proteins.

Fruit, a propagative organ, is an important source of organic acids for humans. The constitution and ratio of organic acids varies between species and even cultivars, e.g., malate is the main organic acid in apple fruit [17], while citrus fruit are rich in citrate [7]. In fruit tissue, most research has focused on organic acid biosynthesis and degradation. In comparison, transportation of organic acids is poorly understood. So far, most of the known transport proteins characterized in fruit have been those involved in malate transportation, including an *AttDT* and four *AtALMT9* homologous in grape berries [14, 18–20] and two *ALMT*-like genes discovered in apple [21], which localize in the vacuole membrane and function as malate transport proteins. Studies on transportation of other organic acids, such as citrate, lag behind those on malate. So far, only one citrate transporter has been identified, *CsCit1*, and was reported to be involved in citrate transport in citrus fruit [22]. Thus, it is important to characterize more citrate metabolism related transport proteins for further understanding of the underlying mechanism for organic acid manipulation.

Citrate is abundant in citrus fruit, a most widely grown and economically important fruit tree crops in the world [23], which includes sweet orange (*Citrus sinensis*), mandarin (*Citrus reticulata*), grapefruit (*Citrus paradisi*), pummelo (*Citrus grandis*) and lemon (*Citrus limon*) [24]. The citrate begins to accumulate during the early phase of fruit development, and decreases remarkably towards maturity in most citrus cultivars, including mandarin and sweet orange. In some other cultivars, however, a high citrate content is maintained in mature fruit, such as Gaocheng hybrid [25] and lemon, which may reduce fruit quality and commodity value. In addition, citrate level in fruit can be affected by the environment during growth and postharvest treatments, e.g., the environments in two habitats have significant effects on citrate levels in navel orange fruits [7], and hot air treatment of fruits was found to have significant effects on citrate content [8]. These variations in citrate highlight citrus as an outstanding material for research on the factors responsible for controlling citrate content.

In the present study, a systematic RNA-Seq strategy was applied to investigate the mechanisms for regulation of citrate degradation in citrus fruit. Three developmental stages from two citrus cultivars, Gaocheng (*Citrus sp*., ‘GC’) and Satsuma Mandarin (*Citrus unshiu* Marc., ‘SM’), with different acidity, were used as materials. Some transport proteins were characterized as citrate degradation related, and these were further tested with a batch of hot air-treated fruit samples, in which citrate degradation occurred faster than in control fruit. The possible roles of these transport proteins in citrate degradation are discussed.

## Materials and Methods

### Plant Material and Treatments

Two citrus cultivars, Gaocheng (*C*. *sp*.) with high acidity and Satsuma mandarin (*C*. *unshiu* Marc.) with low acidity, were collected from orchards belonging to Zhejiang Citrus Research Institute located in Taizhou, Zhejiang, China. Nine fruit, with uniform size, were collected from three different trees for each cultivar at each sampling point. Fruit size and color were measured to monitor the developmental stages. Fruit of six developmental stages (S1–S6) were collected at 60, 75, 90, 120, 150 and 180 days after anthesis in ‘SM’, while they were collected at 75, 90, 105, 135, 165 and 195 days after anthesis in ‘GC’. All the fruits were transported to the laboratory on the same day, the flesh samples were taken and immediately frozen in liquid nitrogen and stored at-80°C.

In order to accelerate the degradation of citrate in ‘GC’ fruit, a hot air treatment was applied. The mature ‘GC’ fruit were subjected to the following treatments: (1) Hot air treatment (HT): the fruit were kept in a chamber at 40°C, > 90% relative humidity (RH) for two days followed by storage at 20°C, 85–95% RH; (2) Control: the fruit were stored at 20°C, 85–95% RH throughout. The fruit were sampled at 0, 2, 10, 20 days after storage. The initiation of the treatments is referred to 0 d in storage. Each sample consisted of nine fruit for each treatment, separated into three replicates with three fruit for each. This batch of fruit was used to test the relationship between candidate transport proteins and citrate degradation.

### Titrable acidity (TA) and Total Soluble Solids (TSS) Measurement

TA of juice sacs was titrated with 0.1 N NaOH to the end point at pH 8.2 according to the method described by Chen et al. [7]. TSS was measured using a digital hand-held refractometer (PR101-α, Atago, Japan). Three drops of juice from one segment were measured, and the procedure was repeated twice per fruit with nine single fruit replicates.

### Metabolites Analysis by GC-MS

The metabolites among the samples were measured according to previous researches [26, 27]. Mixed sample of 0.3 g were ground in liquid nitrogen and extracted with 3 ml of methanol. The mixture was extracted at 70°C for 15 min, and centrifuged at 10,000 g. The upper phase was removed and stored at-80°C until analysis. Aliquots of 100 μl of the upper phase were dried in vacuum and 10 μl ribitol (0.2 mg/ml) was included in each sample as an internal standard. The residue was dissolved in 40 μl of 20 mg/ml pyridine methoxyamine hydrochloride, and incubated for 1.5 h at 37°C. The sample was then treated with 60 μl Bis (trimethylsilyl) trifluoroacetamide (1% trimethylchlorosilane) for 30 min at 37°C.

A volume of 1 μl for each sample was absorbed with a split ratio 25:1 and injected into the gas chromatograph fitted with a fused-silica capillary column (30 m × 0.25 mm i.d., 0.25 μm DB-5 MS stationary phase). The injector temperature was 250°C and the helium carrier gas had a flow rate of 1.0 ml/min. The column temperature was held at 100°C for 1 min, increased to 184°C with a temperature gradient of 3°C/min, increased to 190°C at 0.5°C/min, held for 1 min, increased to 280°C at 15°C/min and then held for 5 min. The significant MS operating parameters were as follows: ionization voltage was 70 eV, ion source temperature was 230°C and the interface temperature was 280°C.

Identification of metabolites was preliminarily based on comparison with the retention time (RT) of the available authentic standards and further identification was based on matching mass spectral fragmentation patterns with those stored in the NIST/EPA/NIH Mass Spectral Library (NIST-08) of the GC-MS data systems. Percentages of the identified compounds were obtained by normalizing the data using the internal standard method. The absolute amounts of the main organic acids and sugars were determined by comparison with calibration standard curves.

### RNA Isolation and RNA-Seq

Total RNA was extracted from frozen flesh following our previously published method [28]. After removal of genomic DNA by RNase-free DNase I (Fermentas), the total RNA was used for RNA-seq and real-time PCR. For RNA-Seq, the RNA was extracted from mixed samples of nine fruit from each developmental stage, S1 (the first stage), S3 (highest acid stage) and S6 (the mature stage), and sequenced using Illumina HiSeq 2000 by Novogene Bioinformatics Institute (Beijing, China). The clean reads were filtered from the raw reads by removing low-quality reads containing ambiguous nucleotides or adaptor sequences. Gene expression levels were calculated by the RPKM method [29]. If there was more than one transcript for a gene, the longest one was used to calculate its expression level and coverage. Statistical analysis was conducted to summarize the number of clean reads that aligned to the recently released *Citrus clementina* reference genome (http://www.citrusgenomedb.org/species/clementina/genome1.0). Referring to the published results [30], an R package (DEGseq) was used to identify differentially expressed genes. For the above methods, the P-values calculated for each gene are adjusted to Q-values for multiple testing corrections. We used Q-value < 0.005 and the absolute value of log_2_Ratio > 1 as the threshold to judge the significance of the difference in gene expression.

### RT-PCR and Quantitative Real-time PCR (qRT-PCR)

First-strand cDNA was synthesized from 1.0 μg DNA-free RNA using RevertAid Premium Reverse Transcriptase (Fermentas). The PCR mixture (10 μl total volume) comprised 2 μl of LightCycler FastStart DNA Master^plus^ SYBR Green I Master Mix (Roche), 0.5 μl of each primer (10 μM), 1 μl cDNA and 6μl water. qRT-PCR was performed on a LightCycler 1.5 instrument (Roche), initiated by 5 min at 95°C, then followed by 45 cycles of 95°C for 10 s, 60°C for 10 s and 72°C for 15 s, and completed with a melting curve analysis program. No-template controls and melting curve analyses were included in every reaction. The actin gene was included as an internal control, using ACT-F (5’-CATCCCTCAGCACCTTCC-3’) and ACT-R (5’-CCAACCTTAGCACTTCTCC-3’) as primers [31]. Primers for the qRT-PCR are list in Table 1.

**Table 1:** Primers used in this study.

Gene	Forward primer (5′ to 3′)	Reverse primer (5′ to 3′)
*CitACO3* ^a^	GCATGAGGCATGAGGATTC	TTGGCCAAAAGAAAAATGAA
*CitGS2* ^a^	TGAGCATCGATGACGAAGAA	TGGCAAGGAACAAGTTCAAA
*CitGDU1* ^a^	ATCTTGGCTTGTTCGTACTGG	TTGTCACCGCTTTCAATGTCT
*CitAL-MT* ^a^	CGACTCCAGGGCAGCATAG	ACTTTCAGCATCGGCTTGTT
*CitCHX* ^a^	CATTTCACAAGCACCAGAGGTT	GCTTCCACCAAGACCACGAT
*CitDIC* ^a^	GTCGCTCGTTGCTCCAGAAC	TGGGCGTACAACTGGCTTC
*CitGDU1* ^b^	CATGGGTGTTAAAGGATTTGTTG	TCAAAACTCTTTGAGCAGCTTC
*CitAL-MT* ^b^	GAAGCAGAGTGTGCAAACAGG	CTCAAAAACTTGGTTGAACTAATAGAC
*CitCHX* ^b^	TGATTAATCGTTGCAAAAACATG	TCCACTTCCAAGTTGAAGCC

^a^ Primers used to analyze gene expression patterns by quantitative real-time PCR (qRT-PCR).

^b^ Primers used to amplify the ORFs.

### Transient Expression in Tobacco

Transient expression was performed with tobacco (*Nicotiana tabacum*) leaves as previously reported [32, 33]. Open reading frames (ORF) of *CitCHX* and *CitDIC* were amplified with the primers described in Table 1 and recombined into pGreenII 0029 62-SK vector. The constructs were electroporated into *Agrobacterium tumefaciens* GV3101 (MP90) individually, and were infiltrated into the abaxial leaf surface for the induction of citrate degradation. A strain containing empty pGreenII 0029 62-SK vector served as a negative control. Target genes and negative controls were included in two sides of the same leaves. Every treatment was carried out with six biological replicates in six different tobacco plants.

### Statistical Analysis

Standard errors and figures were drawn using Origin 8.0 (Microcal Software Inc., Northampton, MA, USA). Least significant difference (LSD) at the 0.05 level was calculated by DPS 7.05 (Zhejiang University, Hangzhou, China).

## Results and Discussion

### Citrus Fruit Acidity was Mostly Related to Citrate Degradation

**Fig 1:**
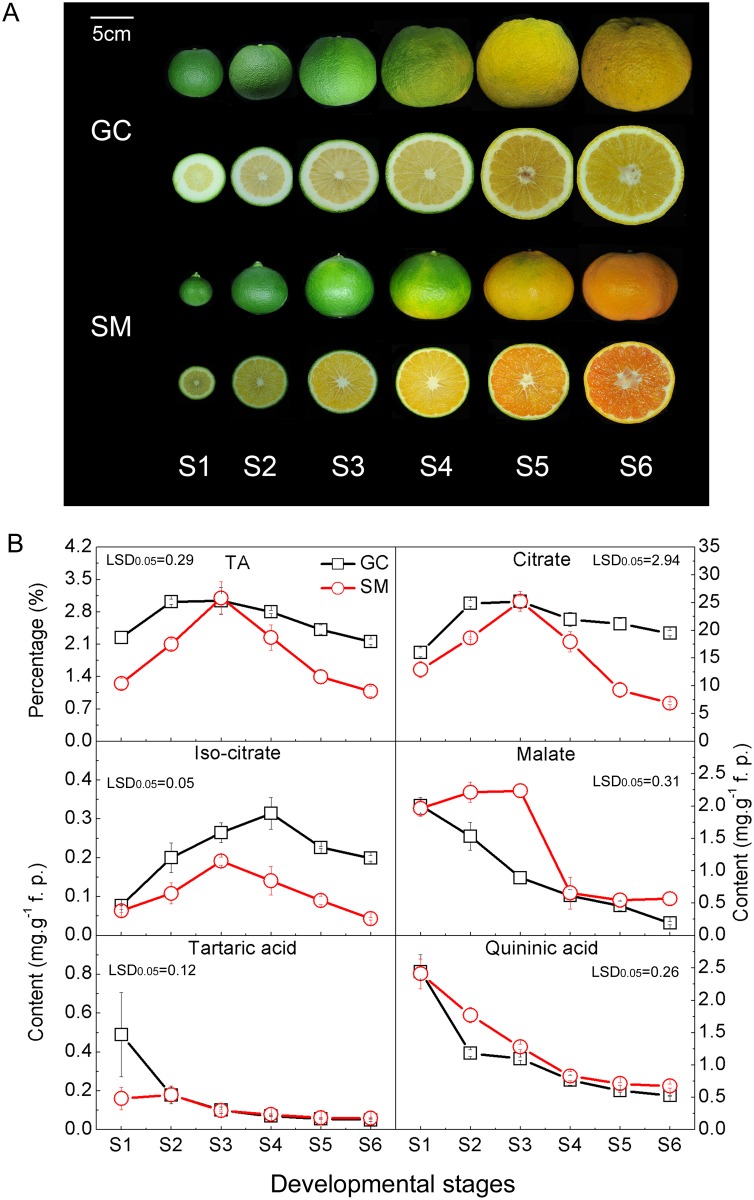
Variation in organic acids content in Gaocheng (‘GC’) and Satsuma mandarin (‘SM’) fruits during development. **(A)** ‘GC’ and ‘SM’ fruits at different developmental stages. The bar indicates 5cm. **(B)** Titratable acidity (TA) and main organic acids at each developmental stage of ‘GC’ and ‘SM’ fruits. The error bars represent the standard errors. The patterns and absolute contents of citrate indicate that it made a dominant contribution to the different acidity of ‘GC’ and ‘SM’ fruit. LSDs represent least significant differences at the 0.05 level.

**Fig 2:**
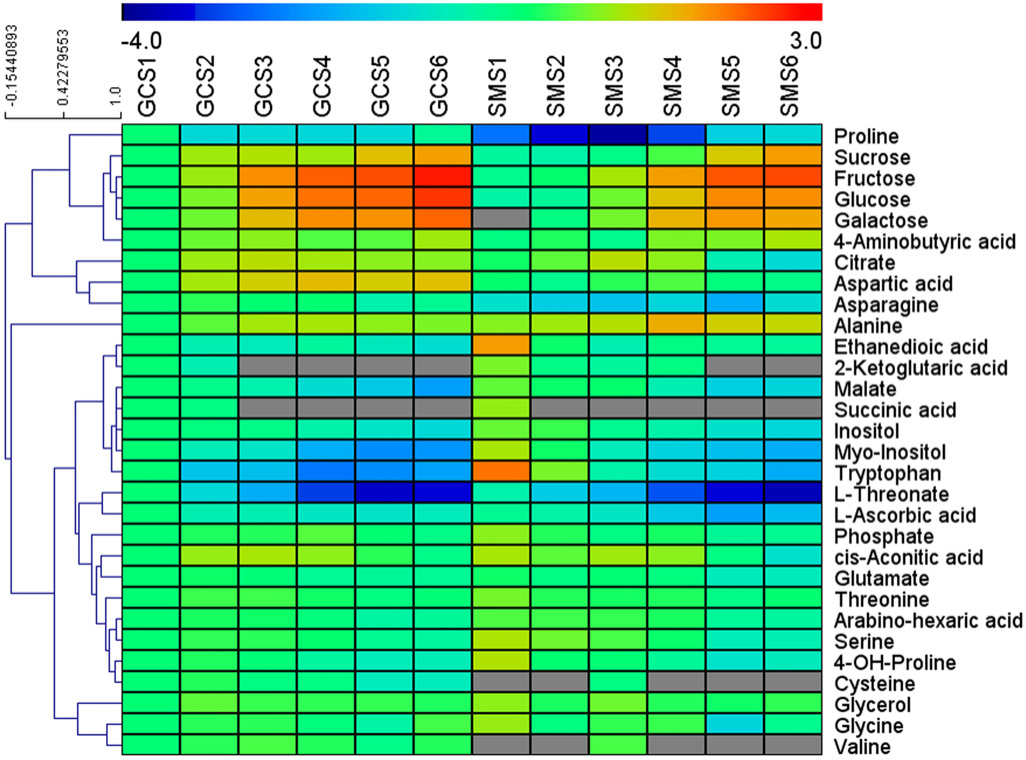
Cluster analysis of metabolites analyzed by GC-MS during ‘GC’ and ‘SM’ development and ripening. GCS1 to GCS6 and SMS1 to SMS6 represent the six developmental stages of Gaocheng and Satsuma mandarin respectively. The graph shows the relative level of each metabolite relative to its amount at GCS1. Normalized values are shown on a color scale (shown on the top of the figure), which is proportional to the content of each identified metabolite, and the grey color indicates not detected. Mean values of 3 independent determinations for each stage were expressed as the ratio between log2 and GCS1 using the MeV 4.8.1 software.

Similar TA contents were found for the two cultivars at S3, but the TA value in ‘SM’ fruit decreased faster than that in ‘GC’ fruit from S4 onwards (Fig. 1). By using GC-MS, 33 metabolites were identified and their levels relative to those detected at stage S1 of ‘GC’ were measured and clustered using MeV 4.8.1 software (Fig. 2). Among the 33 metabolites, sugars displayed the most pronounced and continuous increment towards maturity for the two cultivars. The patterns of change in various organic acids measured during citrus fruit development and ripening were diverse. 2-ketoglutaric acid, succinic acid, quininic acid, tartaric acid, L-ascorbic acid and ethanedioic acid levels were high in S1 and continuously decreased during development until maturity. Malate levels peaked at S3 and then fell rapidly in ‘SM’, but were decreasing continuously in ‘GC’ from S1 to S6. On the other hand, citrate and isocitrate showed a significant increase during the early developmental stages (from S1 to S3) and then decreased rapidly in ‘SM’ fruit, while remaining constant in ‘GC’ fruit. In addition, 10 amino acids were also detected in both ‘GC’ and ‘SM’ fruit, including GABA, aspartic acid, asparagine, alanine, tryptophan, glutamate, serine, cysteine, valine and glycine (Fig. 2). Variations in most of the compounds were consistent with the previous research on metabolites profiling in citrus fruit [34].

The absolute quantitative determination indicated that citrate is the most abundant acid in both ‘GC’ and ‘SM’ fruits. The patterns and absolute contents of citrate (note the differences in scales in Fig. 1B), which is consistent with the changes in TA content, indicated that it made a dominant contribution to the different acidity of ‘SM’ and ‘GC’ fruit. The two fruit cultivars accumulated almost the same citrate content at S3 (about 25 mg/g), indicating that there was little difference in the citrate synthesis and accumulation. This is consistent with previous reports that the citrate content in mature fruit was not regulated by synthesis [7, 35]. The two cultivars, however, exhibited quite different patterns of citrate degradation as shown in Fig. 1B. From S3 to S6, citrate content decreased remarkably in ‘SM’, but was maintained at a high level in ‘GC’ until maturation, suggesting that there was an obstacle in the citrate degradation pathway during ripening in ‘GC’.

### Transcriptome Analysis of Differentially Expressed Genes (DEGs)

**Fig 3:**
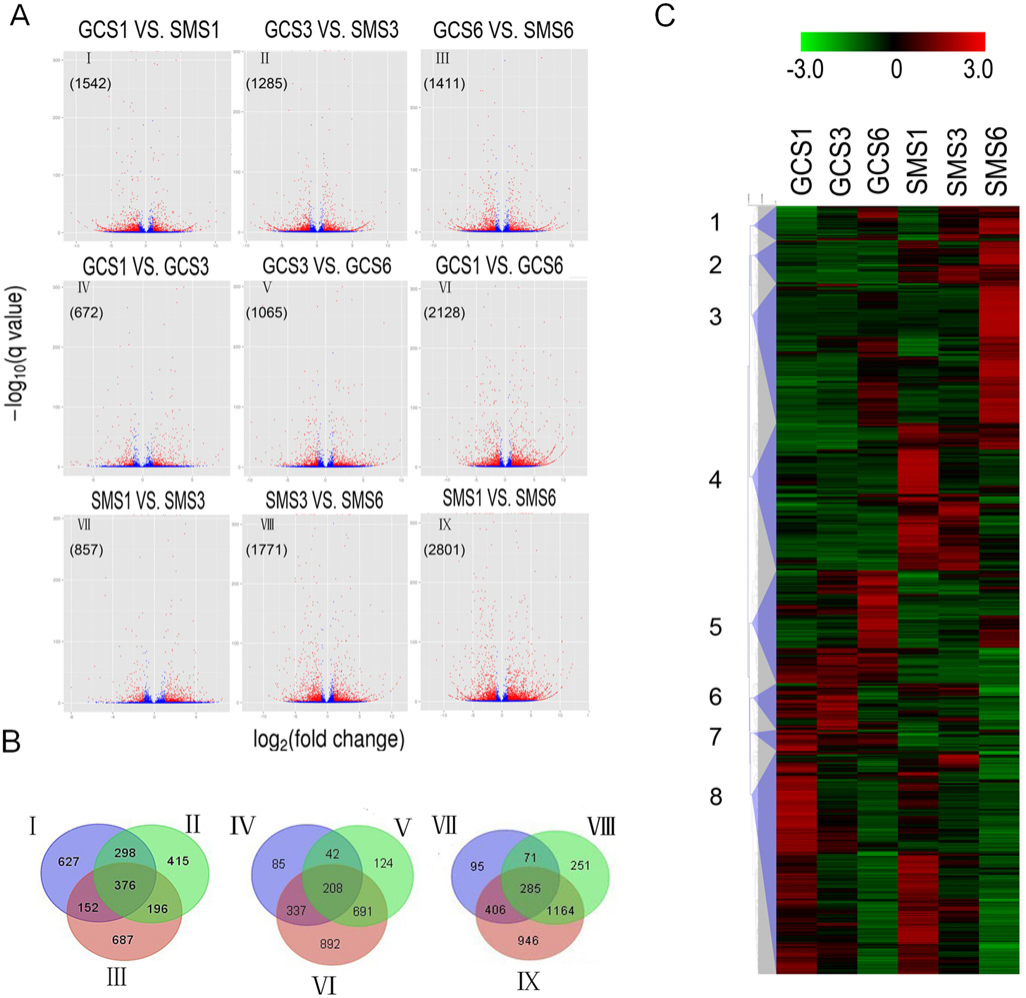
Transcriptome analysis of differentially expressed genes (DEGs) at different developmental stages of Gaocheng and Satsuma mandarin. **(A)** Volcano plot showing genes differently expressed between different libraries. Q-value < 0.005 and the absolute value of log_2_Ratio > 1 were used as the threshold to judge the significance of the difference in gene expression. Red plots represent genes with significantly different expression; blue plots represent those showing no significant difference. **(B)** Venn diagram of relationship between DEG groups. The numbers indicate the DEG number in each DEG group shown in Fig. 2. **(C)** Hierarchical clustering of all differentially expressed genes, classified into 8 sub-clusters. The color (from green to red) represents gene expression intensity from low to high. GCS1, GCS3 and GCS6 represent stages S1, S3 and S6 of Gaocheng, while SMS1, SMS3 and SMS6 represent stages S1, S3 and S6 of Satsuma mandarin.

To obtain a general overview of metabolism in citrus fruit, six libraries (GCS1, GCS3, GCS6, SMS1, SMS3 and SMS6) were constructed for RNA-Seq. Each library produced more than 2G clean bases with a Q20 percentage over 97% (Table 2). The clean reads were aligned to the recently released *C*. *clementina* reference genome. Of the clean reads, more than 82% matched either to a unique or multiple genomic locations. From all the libraries, 25,779 genes were obtained in total, including 1,246 new transcripts (S1 Dataset). One of the primary goals of the transcriptome study was to identify variations between different libraries. The results indicated that these variations ranged from 672 to 2,801 DEGs, based on RPKM values (Fig. 3A). The relationships between different DEG groups from Fig. 3A were displayed as Venn diagrams, and the results indicated that 376 DEGs were shared by all developmental stages, while 627, 415 and 687 DEGs were specific to S1, S3 and S6, respectively (Fig. 3B). When all libraries were combined together, a total of 4,866 DEGs, which predicted eight groups (1 to 8) using hierarchical clustering methods, were defined according to the expression profiles (Fig. 3C). When compared to ‘GC’, genes in ‘SM’ showed up-regulation in sub-clusters 1, 2, 3 and 4 and down regulation in sub-clusters 5, 6, 7 and 8. Within these groups, 648 genes were highly associated with citrate content (S2 Dataset). In the past few years, RNA-seq has been widely used in citrus based on the published genome, including research on lycopene accumulation [36], male sterile cybrids [37], flower development, disease [38], etc. Our present data provided additional transcriptome information concerning ‘GC’ and ‘SM’ development, which provides the basis for further understanding and manipulation of citrus fruit development.

**Table 2:** Throughput and quality control of RNA-Seq data from Gaocheng and Satsuma mandarin libraries.

Sample^a^	Clean bases	Q20(%)^b^	Total mapped(%)^c^	Uniquely mapped(%)	Splice reads(%)^d^
GCS1	2.38G	98.53	84.98	83.28	28.10
GCS3	2.61G	97.22	82.09	79.02	27.62
GCS6	2.27G	98.52	85.42	82.75	29.01
SMS1	2.80G	98.60	88.00	85.64	27.44
SMS3	2.61G	98.58	88.60	86.28	28.67
SMS6	2.72G	98.54	88.65	87.30	29.28

^a^ GCS1. GCS3 and GCS6 represent stages S1, S3 and S6 of Gaocheng, while SMS1, SMS3 and SMS6 represent S1, S3 and S6 of Satsuma mandarin.

^b^ The percentage of sequences with sequencing error rate lower than 1%.

^c^ The percentage of clean bases mapped to the reference genome.

^d^ The percentage of sequences aligned to two transcripts.

### Citrate Metabolism

**Fig 4:**
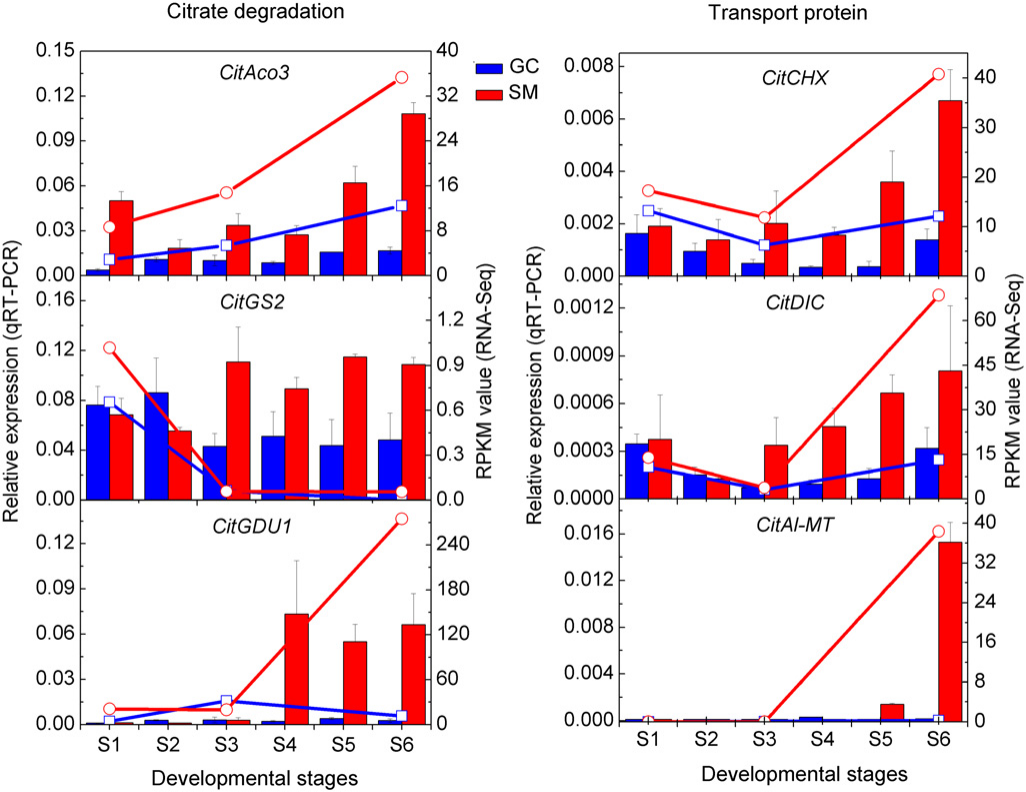
Transcript expressions of genes related to citrate degradation and transport as measured by RNA-Seq and qRT-PCR. Lines represent expression determined by RNA-Seq in RPKM value (right axis), while histograms represent transcript expression determined by qRT-PCR (left axis). The error bars represent the standard errors.

No differentially expressed *CitCS* and *CitPEPC* was observed in either cultivar (Table 3), which indicated that *CitCS* and *CitPEPC* were not responsible for differences in acidity in citrus. This is consistent with previous reports [7, 35, 39]. As for possible mechanisms for citrate degradation, since no difference in *CitGAD* and *CitACL* expression between the two cultivars was observed, this suggested that the GABA and acetyl-CoA pathways are not the main degradation pathway during fruit development in citrus (Table 3). The up-regulation of *CitAco3* and *CitGS2* in ‘SM’ during citrate degradation (Fig. 4) indicates that citrate is most likely degraded through the glutamine pathway in ‘SM’, which is similar to previous conclusions [7]. Furthermore, we also found that *CitGDU1* (Ciclev10009695m) was up-regulated (Fig. 4) towards maturity in ‘SM’. GDU was reported to transport glutamine out of the cytoplasm [40, 41] and there is a possibility that the activity of *CitGDU1* maintains the catalytic rate of glutamine synthase, ensuring the continued degradation of citrate. Thus, the up-regulation of the *CitAco3-CitGS2-CitGDU1* cascade may be the main reason for citrate degradation during ‘SM’ fruit development.

**Table 3:** Differentially expressed genes related to citrate metabolism in citrus fruit.

Enzyme name	Abbreviation	All ^a^	DEGs ^b^	Serial number	RPKM value					
					GCS1	GCS3	GCS6	SMS1	SMS3	SMS6
Phosphoenolpyruvate carboxylase	*CitPEPC*	7	0							
Citrate synthase	*CitCS*	2	0							
Aconitase	*CitAco*	3	1	Ciclev10007338m	2.88	5.41	12.46	8.66	14.79	35.32
Isocitrate dehydrogenase	*CitIDH*	5	1	Ciclev10001314m	327.50	339.60	573.96	171.57	234.33	203.90
Glutamate decarboxylase	*CitGAD*	5	0							
GABA aminotransferase	*CitGABA-T*	1	0							
Glutamine synthetase	*CitGS*	2	1	Ciclev10011785m	109.13	93.25	240.30	123.50	123.47	368.99
ATP-citrate lyase	*CitACL*	4	0							
Phosphoenolpyruvate carboxykinase	*CitPEPCK*	9	4	Ciclev10000510m	223.70	508.18	770.78	131.82	249.53	382.68
				Ciclev10005624m	173.07	932.02	185.42	233.60	374.34	74.67
				Ciclev10020693m	55.28	97.08	6.75	63.40	59.33	3.54
				Ciclev10025088m	15.64	8.41	49.12	36.21	38.23	232.73

a, All gene members.

b, Differentially expressed members.

### Characterization of Transport Proteins Involved in Citrate Degradation

In addition to the citrate metabolism, close attention was also paid to citrate-related transport proteins, which have not been well studied previously. Most of the citrate content of fruit is found in the vacuole, which occupies 90% of the volume of most mature fruit cells [42]. However, citrate is degraded in the cytosol, and efflux of citrate probably occurs through specific carriers or transporters. According to previous research, the highest expression and the greatest amount of protein of the identified vacuolar citrate transporter, *CsCit1*, coincided with the developmental stage at which the vacuolar citrate content began declining with the concomitant increase in vacuolar pH [22]. However, according to RNA-Seq data, the changes in expression pattern of *CsCit1* (Ciclev10014770m) was not consistent with the pattern of citrate degradation (Data not shown), which indicated that other transport proteins might be involved in citrate degradation and the critical transport protein may vary among citrus cultivars as well.

From our data, a malate channel gene (*CitAl-MT*, Ciclev10019573m) was significantly up-regulated at S6 in ‘SM’, but in ‘GC’ fruit, the expression level of this gene was very low at all developmental stages (Fig. 4). A dicarboxylate carrier gene (*CitDIC*, Ciclev10001822m) was significantly up-regulated in ‘SM’ during ripening, and its expression steadily increased from S3 to S6 (Fig. 4). The cation/H^+^ exchanger gene (*CitCHX*, Ciclev10018903m) was also significantly up-regulated in ‘SM’ during ripening, and showed a similar expression trend with *CitDIC* (Fig. 4). From previous reports, *DIC* was reported to transport both tricarboxylates such as citrate and dicarboxylates such as succinate, malate and fumarate across the plasma membrane in human cells [43], and it was also used to transport malate to operate the C_4_ photosynthetic pathway in plants [20]. To our knowledge, citrate transport occurs by facilitated diffusion [44], possibly through the malate channel [45, 46], suggesting there is a possibility that *CitDIC* is involved in citrate degradation in citrus fruit. In addition, the expression of *CitCHX* (׀R׀ = 0.93) and *CitDIC* (׀R׀ = 0.92) were highly correlated with the citrate content variation. It is possible that *CitCHX* is co-regulated with *CitDIC*, leading to a rapid degradation of citrate in the fruit cell cytoplasm.

### Transport Proteins Involved in Hot Air Stress-activated Citrate Degradation

**Fig 5:**
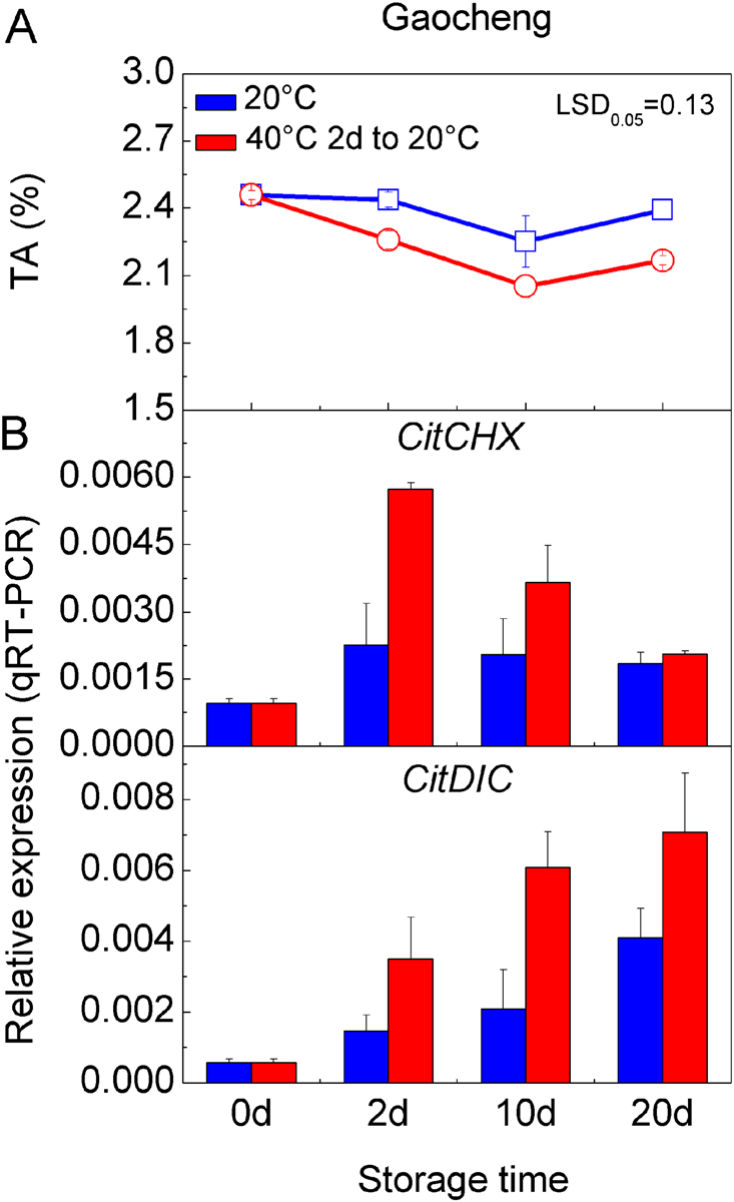
Expression of transport-related genes in Gaocheng fruit treated with hot air. **(A)** The TA content at each storage stage of development Gaocheng fruit treated with 40°C hot air for 2 days; **(B)** Expression of *CitCHX* and *CitDIC* at each stage. The error bars represent the standard errors. LSD represents least significant differences at 0.05 levels.

To perturb the obstacle to citrate degradation obstacle in ‘GC’ fruit, we applied hot air treatment to mature fruit, which causes an accelerated reduction in citrate content [8]. Fig. 5A shows that the TA content was substantially lower in hot air-treated fruit, reduced by 7%-9%, suggesting this was also effective on ‘GC’ fruit. Accompanying this marked reduction, the expressions of the two transport-related genes were induced by 2-fold under hot air treatment. An increase in *CitCHX* mRNA was also stimulated by the hot air treatment immediately and this subsequently decreased steadily, but still remained at high levels during storage compared to the control. *CitDIC* mRNA increased continuously during storage in both treatments and the control, although the level during hot air treatment was significantly higher than that in the control (Fig. 5B). It has been reported that *AtCHX17* expression was strongly induced by salt stress and external acidic pH [47], and that *DIC* was involved in aluminum tolerance [48]. From our results, *CitCHX* and *CitDIC* also responded to heat stress. Hot air treatment accelerated the decrease in citrate content in citrus fruit significantly, but had less effect on the content of malate [8]. Thus, *CitCHX* and *CitDIC* are most likely to be involved in hot air stress activated citrate degradation in citrus fruit.

The *CitCHX* mRNA was activated immediately after hot air treatment, while the *CitDIC* mRNA increased after the expression peak of *CitCHX* mRNA. It has been reported that CHX is responsible for H^+^ efflux from the vacuole and results in different vacuole acidity [49]. The cation/H^+^ antiporter, which mediates an electroneutral exchange, has no effect on Δψ. In the case of the cation/H^+^ antiport, there is an additional effect on pH due to protons leaving the vacuole, thus, maintaining the citrate^2-^ efflux from vacuole to cytoplasm. According to our results, there is a possibility that the *CitCHX* mediated a large H^+^ flux from the vacuole, and maintained the transport activity of the *CitDIC* protein.

### Transient Over-expression in Tobacco Leaves

**Fig 6:**
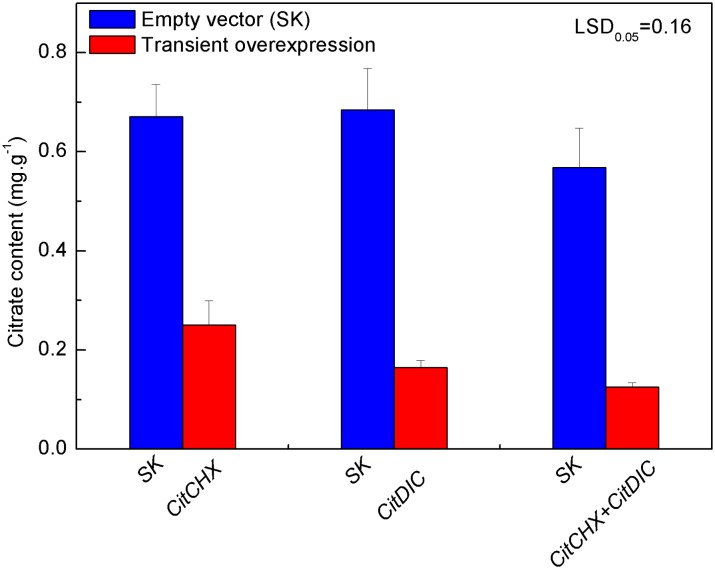
Citrate accumulation activated by *CitCHX* and *CitDIC*, separately or coordinately, in *Nicotiana tabacum* leaves. Citrate content was measured on the fifth day after infiltration. *CitCHX* and *CitDIC* were driven by the CaMV 35S promoter. SK represents empty vector. Error bars represent the standard errors from six biological replicates. LSD represents least significant differences at the 0.05 level.

Most interestingly, transient over-expression of *CitCHX* and *CitDIC*, separately or coordinately, caused a major decrease of citrate (Fig. 6), and the citrate content was reduced by 62%, 75% and 78% following *CitCHX*, *CitDIC* and *CitCHX* plus *CitDIC* treatments, respectively, as compared with the effect of an empty vector. This indicated the expression of only one of these genes is sufficient to cause a loss of stored citrate. Although the expression of *CitCHX* plus *CitDIC* also caused lower citrate content, there was no significant difference between the single and combined treatments. The relationship between transport proteins and citrate content has been reported in other plants [9, 11, 12], but did not include a study of the above-mentioned transport proteins. For instance, a higher proportion of citrate was discovered in *AttDT*::*tDNA* plants when compared to wild-type, and the increment in cellular citrate in knock-out lines was shown to be mainly due to a higher vacuolar concentration of citrate, whereas the cytosolic citrate concentrations were similar in both plant genotypes [50]. It is proposed that citrate degradation was promoted by coordinated action of these two transport proteins. However, it is not clear whether these genes can participate in the transport process directly or regulate the process by unknown mechanism.

## Conclusions

The difference in acidity in mature fruit of ‘GC’ and ‘SM’ citrus was mainly caused by the different degradation rates of citrate. RNA-Seq analysis of ‘GC’ and ‘SM’ provided substantial transcriptome information during fruit development. Two steps were proposed for citrate degradation in citrus fruits. Firstly, the citrate is transported out of the vacuole, and the transport-related genes, *CitCHX* and *CitDIC*, are possibly involved in this process. The exported citrate is then available to be metabolized by the *CitAco3-CitGS2-CitGDU1* cascade during fruit development. In addition, *CitCHX* and *CitDIC* are also involved in hot air stress activated citrate degradation in citrus fruit. However, the underlying mechanism of how these transport related genes participate in the citrate degradation process needs further research.

## Supporting Information

S1 Dataset The RPKM value of gene expression annotated from RNA-Seq data. (XLS)

S2 Dataset The correlation analysis of variation of citrate content and expression trends of genes in sub-cluster I, II and III in Fig. 2c. (XLS)
